# Impact of bacterial vaginosis on sexually transmitted viral infections: a bacterial point of view

**DOI:** 10.1093/femsre/fuaf039

**Published:** 2025-08-28

**Authors:** Celia Segui-Perez, Marleen Y van Smoorenburg, Anna E Maranus, Teunis B H Geijtenbeek, Karin Strijbis

**Affiliations:** Section Infection Biology, Division of Infectious Diseases and Immunology, Department of Biomolecular Health Sciences, Faculty of Veterinary Medicine, Utrecht University, Yalelaan 1, 3584 CL, Utrecht, the Netherlands; Amsterdam UMC, location University of Amsterdam, Department of Experimental Immunology, Meibergdreef 9, Amsterdam, the Netherlands; Amsterdam institute for Immunology and Infectious Diseases, Infectious Diseases, Amsterdam, the Netherlands; Section Infection Biology, Division of Infectious Diseases and Immunology, Department of Biomolecular Health Sciences, Faculty of Veterinary Medicine, Utrecht University, Yalelaan 1, 3584 CL, Utrecht, the Netherlands; Amsterdam UMC, location University of Amsterdam, Department of Experimental Immunology, Meibergdreef 9, Amsterdam, the Netherlands; Amsterdam institute for Immunology and Infectious Diseases, Infectious Diseases, Amsterdam, the Netherlands; Section Infection Biology, Division of Infectious Diseases and Immunology, Department of Biomolecular Health Sciences, Faculty of Veterinary Medicine, Utrecht University, Yalelaan 1, 3584 CL, Utrecht, the Netherlands

**Keywords:** vaginal microbiota, bacterial vaginosis, *Gardnerella vaginalis*, *Hoylesella timonensis*, *Prevotella timonensis*, *Prevotella bivia*, *Fannyhessea vaginae*, *Atopobium vaginae*, *Mobiluncus mulieris*, *Sneathia amnii*, HIV-1

## Abstract

Bacterial vaginosis (BV) is a complex polymicrobial vaginal infection that affects a large percentage of women during different stages of life including the reproductive age. In a healthy vaginal environment, the epithelium is colonized by protective *Lactobacillus* species that make up 90%–95% of the total vaginal microbiota. BV is characterized by a reduction of lactobacilli and a concurrent increase in diverse anaerobic bacteria, including *Gardnerella vaginalis, Prevotella bivia, Hoylesella timonensis*, and *Fannyhessea vaginae*. BV is associated with an increased risk of infertility, preterm birth, and a higher susceptibility to sexually transmitted infections (STIs), including Human Immunodeficiency Virus type-1 (HIV-1). This review examines the contribution of individual pathogenic bacteria to the development of BV and the resulting effects on susceptibility to STI. The impact of the different key bacterial virulence factors, such as secreted proteins, biofilm formation, and inflammatory potential on subsequent viral infection are discussed. While antibiotics are commonly prescribed to treat BV, recurrence rates are high, and antimicrobial resistance among BV-associated bacteria is increasingly reported. Understanding the mechanisms underlying BV and the impact of specific bacteria and their virulence factors on viral infections can improve preventive strategies and open up novel therapeutic applications.

## Teaser

Review of the individual bacterial vaginosis-associated bacteria, their unique virulence factors, and their impact on viral infection, with an emphasis on the molecular mechanisms of microbe–host interactions in the vaginal mucosa.

## Introduction

The cervicovaginal epithelium consists of a stratified squamous nonkeratinized epithelium covered in cervicovaginal mucus (CVM) and cervicovaginal fluid (Lacroix et al. 2020). This gel-like mucosa layer serves as a protective barrier against infectious microorganisms, lubricates the vaginal epithelium, and harbors the vaginal microbiome (Allen et al. 1982). The vaginal microbiome is influenced by several factors, including hormonal levels (Farage et al. 2010), race (Sun et al. 2022), number of sexual partners (Nelson et al. 2012, Ma 2022), contraceptive use (Vodstrcil et al. 2017, Achilles et al. 2018), the use of sex toys, and vaginal lubricant (Laniewski et al. 2019).

In healthy women, the vaginal microbiome is dominated by *Lactobacillus* species including *L. crispatus, L. gasseri, L. jensenii*, and *L. iners*. Usually, women are colonized by a single dominant *Lactobacillus* species that accounts for ~90%–95% of the total microbial community (Auriemma et al. 2021: 2, Pendharkar et al. 2023). Under healthy conditions, the lactobacilli produce lactic acid, which maintains the vaginal pH at ∼4 (O’Hanlon et al. 2013, Ñahui Palomino et al. 2017). In addition to lactic acid (Boris and Barbés 2000, O’Hanlon et al. 2011), lactobacilli also produce antimicrobial compounds, such as hydrogen peroxide (Sgibnev and Kremleva 2015), bacteriocins (Stoyancheva et al. 2014), and arginine deaminase enzymes (Rousseau et al. 2005), that all suppress the growth of anaerobic bacteria. Additionally, lactobacilli strongly adhere to the vaginal epithelium, which serves as a protective mechanism as thereby attachment of anaerobic bacteria is limited (Edelman et al. 2012, Leccese Terraf et al. 2014, He et al. 2020, Parolin et al. 2021, Qian et al. 2021). Several reviews about the protective roles of vaginal *Lactobacillus* spp. have recently been published (Amabebe and Anumba 2018, Chee et al. 2020, Pendharkar et al. 2023). Overall, the high prevalence of lactobacilli is key for maintaining an acidic vaginal pH and limiting anaerobic bacterial growth.

Bacterial vaginosis (BV) is a polymicrobial infection that occurs when the vaginal microbiome shifts from a *Lactobacillus*-dominated microbiome toward a higher prevalence of facultative or obligate anaerobic bacteria, such as *Gardnerella* spp., *Hoylesella* spp., *Prevotella* spp., *Fannyhessea* spp., *Mobiluncus* spp., *Sneathia* spp., and BV-associated bacteria (BVAB) 1–3 (Fredricks and Marrazzo 2005, Srinivasan et al. 2012, Gao et al. 2022). The Amsel criteria are commonly used to diagnose BV and are based on an increased vaginal pH (>4.5), thinned vaginal discharge, amine odor after addition of potassium hydroxide, and the presence of “clue cells” (Amsel et al. 1983, Mohammadzadeh et al. 2015). The Nugent score test is also used for BV diagnosis, which involves the quantification of Gram-positive rods, such as lactobacilli (healthy bacteria) and Gram-negative or Gram-variable bacteria (associated with BV) (Nugent et al. 1991, Coleman and Gaydos 2018). BV is associated with infertility (Ravel et al. 2021), adverse pregnancy outcomes such as preterm birth (Dingens et al. 2016), and increased susceptibility to sexually transmitted infections (STIs), including Human Immunodeficiency Virus type-1 (HIV-1) (Sha et al. 2005, Borgdorff et al. 2014, Kyongo et al. 2015, Gosmann et al. 2017), Human Papilloma Virus (HPV) (Gillet et al. 2011, Brotman et al. 2014, Li et al. 2020), and Herpes Simplex Virus type-2 (HSV-2) (Cherpes et al. 2005, Nagot et al. 2007). Virulence factors associated with BV include bacterial adherence to the cervicovaginal epithelium, biofilm formation (Patterson et al. 2010, Machado et al. 2013), and expression of cytotoxins (Gelber et al. 2008, Rampersaud et al. 2011) and enzymes, such as mucinases, sulfatases, galactosidases, and prolidases (Howe et al. 1999, Olmsted et al. 2003, Cauci et al. 2005, Moncla et al. 2016). For instance, sialidases are enzymes found at higher levels in women with BV and have been shown to degrade the protective mucus layer (Briselden et al. 1992, Lewis et al. 2013, Plesniarski et al. 2021) and other glycoproteins, such as the secretory Immunoglobulin A (IgA) (Lewis et al. 2012). Moreover, certain anaerobic bacteria can induce proinflammatory responses, resulting in recruitment of immune cells, some of which can be targets for viruses, such as Langerhans cells and T cells for HIV-1 (Libby et al. 2008, Doerflinger et al. 2014).

Numerous studies have reported associations between an altered vaginal microbiome and increased susceptibility to viral infections that affect millions of women worldwide. For HIV-1 infections, BV is associated with HIV-1 prevalence, increased viral load, and genital HIV-1 RNA shedding (Cherpes et al. 2005, Atashili et al. 2008, Borgdorff et al. 2014, Kyongo et al. 2015, Gosmann et al. 2017). A BV-associated vaginal microbiome is also thought to increase the risk of developing HPV-induced cervical cancer (Gillet et al. 2011, Moscicki et al. 2012, Łaniewski et al. 2018). The HSV-2 virus is the main cause of genital herpes and several studies have suggested an association between BV and an increased risk of infection (Cherpes et al. 2005, Kaul et al. 2007, Nagot et al. 2007). It is evident that the polymicrobial anaerobic colonization in BV greatly enhances susceptibility to viral infections, but the underlying mechanisms and the specific roles of individual bacterial spp. are not fully understood. This review summarizes the current knowledge about virulence traits of specific vaginal BV-associated anaerobic bacteria and their impact on virus infection.

## Virulence-associated traits of anaerobic vaginal bacteria

### Gardnerella vaginalis

*Gardnerella vaginalis* is the most extensively studied BV-associated facultative anaerobic bacterium. Within the *G. vaginalis* clade, there is a high strain diversity and also other non-*vaginalis Gardnerella* subspecies can play roles in BV (Tortelli et al. 2021). Staining of *Gardnerella* subspecies can be Gram-variable. Genomic and functional analyses of different *G. vaginalis* strains demonstrated the presence of a range of virulence traits, including adherence to the epithelium, biofilm formation, hemolytic and cytotoxic abilities, immune evasion, and antimicrobial resistance (Yeoman et al. 2010). *Gardnerella vaginalis* can adhere efficiently to the vaginal epithelium and produce a biofilm (Yeoman et al. 2010, Alves et al. 2014) and in this process displace adherent lactobacilli (Machado et al. 2013). Therefore, *G. vaginalis* is considered an important early colonizer that facilitates the subsequent attachment of other anaerobes.

An important virulence factor of *G. vaginalis* is the cytotoxin vaginolysin that is highly expressed in BV-associated *G. vaginalis* strains. Vaginolysin targets CD59, also known as MAC-inhibitory protein, on epithelial cells, erythrocytes, and neutrophils resulting in membrane blebbing and pore formation (Patterson et al. 2010, Yeoman et al. 2010, Randis et al. 2013, Anton et al. 2022). Bacterial membrane vesicles can carry vaginolysin and induce cytotoxicity and proinflammatory responses in vaginal epithelial cells (Shishpal et al. 2020). *Gardnerella vaginalis* strains also encode oxygen-independent coproporphyrinogen III oxygenases (HemN), which are involved in the release and utilization of iron from hemoglobin and myoglobin (Yeoman et al. 2010). Other toxin-related *G. vaginalis* genes include potential lysozyme-like toxins, toxin–antitoxin genes, and an invasion-associated hydrolase (Yeoman et al. 2010). Together, the *G. vaginalis* virulence factors promote a proinflammatory response in cervicovaginal epithelial cells (Libby et al. 2008, Doerflinger et al. 2014, Santos et al. 2018, Anton et al. 2022), induce cellular exfoliation (Lewis et al. 2013, Gilbert et al. 2019), and increase epithelial permeability *in vitro* (Anton et al. 2022, Berard et al. 2023). However, the *in vivo* importance of the different factors remains unclear.

Similar to the vaginal *Lactobacillus* spp., *G. vaginalis* can utilize glycogen, which is an abundant carbon source in the vaginal epithelium (Bhandari and Hill 2023, Jenkins et al. 2023, Navarro et al. 2023, Segui-Perez et al. 2024). In addition, *G. vaginalis* degrades the protective mucus layer through the production of sialidases that cleave terminal sialic acid from mucins (Gilbert et al. 2019, Agarwal and Lewis 2021). *Gardnerella* spp. encode up to four sialidases, NanH1, NanH2, NanH3, and NanH4, (Robinson et al. 2019) that can be highly abundant in clinical BV samples (Hardy et al. 2017, Pelayo et al. 2024). Of these four enzymes, only *Gv*NanH2 and *Gv*NanH3 display high sialidase activity in *in vitro* assays and can effectively remove sialic acids from N-linked and O-linked sialoglycan substrates (Lewis et al. 2013, Robinson et al. 2019, Pelayo et al. 2024). A recent study demonstrated that recombinant sialidases of *G. vaginalis* efficiently desialylate vaginal epithelial glycans, inducing pathways related to cell death, differentiation, and inflammatory responses (Agarwal et al. 2023). The sialidase activity of *G. vaginalis* also plays an important role in cellular invasion (Govinden et al. 2018) and the proteolytic degradation of IgA molecules (Lewis et al. 2012). These studies underscore the significance of sialidase activity as a virulence factor in a wide range of pathogenic processes. In summary, *G. vaginalis* contributes to BV by initial adhesion to the epithelium, degradation of the protective mucus layer, inducing cytotoxicity, promoting inflammation, and compromising epithelial integrity. The main virulence factors of *G. vaginalis* and the bacteria described below are summarized in Table 1.

**Table 1.** Overview of virulence factors associated with individual anaerobic vaginal bacteria.

Bacteria	Adherence/biofilm	Secretion of toxins and other compounds	Glycosidases/impact on mucins	Immune system activation or evasion	Antibiotic resistance
*Gardnerella vaginalis*	Adheres to the cervicovaginal epithelium, can displace adherent lactobacilli (Machado et al. 2013a), and promotes biofilm formation (Patterson et al. 2010, Yeoman et al. 2010, Alves et al. 2014). Encodes genes for fimbriae/pili (Yeoman et al. 2010).	Produces vaginolysin (Gelber et al. 2008), and encodes other toxins, such as lysozyme-like toxins, oxygen-independent oxidases (HemN), several toxin antitoxins genes, and an invasion-associated hydrolase (Yeoman et al. 2010).It causes cytotoxicity in several epithelial cell lines (Patterson et al. 2010, Castro et al. 2015, Anton et al. 2022, Segui-Perez et al. 2024).	Can utilize glycogen (Bhandari and Hill 2023, Jenkins et al. 2023, Navarro et al. 2023, Segui-Perez et al. 2024) and exhibits sialidase activity, degrading cervicovaginal cell sialoglycans (Gilbert et al. 2019, Robinson et al. 2019), and secretory IgA (Lewis et al. 2012).	Induces IL-8, IL-6, and CCL-20 production by cervicovaginal epithelial cells (Libby et al. 2008, Anton et al. 2022, Segui-Perez et al. 2024), and HeLa cells (Santos et al. 2018).	Some strains are resistant to metronidazole and bleomycin (Yeoman et al. 2010, Petrina et al. 2017).
*Hoylesella timonensis*	Adheres to the cervicovaginal epithelium (Ilhan et al. 2020, Segui-Perez et al. 2024). Forms a sparse biofilm and elongates microvilli in endometrial cells (Ilhan et al. 2020).	Does not have cytotoxic activity on cervicovaginal cells (Segui-Perez et al. 2024).	Can utilize glycogen (Jenkins et al. 2023, Segui-Perez et al. 2024) and degrade the vaginal mucus layer due to high sialidase and fucosidase activities (Jenkins et al. 2023, Pelayo et al. 2024, Segui-Perez et al. 2024). Encodes an extensive array of carbohydrate-active enzymes (CAZymes) (Segui-Perez et al. 2024).	Does not induce a proinflammatory response in vaginal epithelial cells (Ilhan et al. 2020, Segui-Perez et al. 2024), but stimulates dendritic cells toward Th1 polarization (van Teijlingen et al. 2020). Increases HIV-1 uptake in Langerhans cells and DCs (Van Teijlingen et al. 2022) and infection in CD4 + T cells (van Teijlingen et al. 2024).	Some strains are resistant to clindamycin (Petrina et al. 2017).
*Prevotella bivia*	Does not adhere to cervicovaginal epithelial cells (Segui-Perez et al. 2024), but forms a sparse biofilm in endometrial cells (Ilhan et al. 2020). Can join a *G. vaginalis*-biofilm (Machado et al. 2013, Castro et al. 2021a).	Produces ammonia (Pybus and Onderdonk 1997), polyamines (Łaniewski and Herbst-Kralovetz 2021), and collagenases (Doust and Mobarez 2004). Does not have cytotoxic activity (Segui-Perez et al. 2024).	Can degrade glycogen (Segui-Perez et al. 2024). Expresses sialidase activity, but does not efficiently degrade the glycocalyx in vaginal epithelial cells (Gilbert et al. 2019, Segui-Perez et al. 2024).	Does not induce a proinflammatory response in cervicovaginal epithelial cells in most studies (Doerflinger et al. 2014, Ilhan et al. 2020, Segui-Perez et al. 2024).	Some strains are resistant to clindamycin and metronidazole (Petrina et al. 2017, Veloo et al. 2018).
*Fannyhessea vaginae*	Does not adhere well to the epithelium on its own. Joins *G. vaginalis* biofilm (Hardy et al. 2016, Castro et al. 2019, 2020).	Does not induce cytotoxicity in ME-180 vaginal epithelial cells(Patterson et al. 2010).	Not described.	Upregulates proinflammatory cytokines TNFα, CCL-20, IL-6, and IL-8 in cervicovaginal epithelial cells (Trama et al. 2007, Libby et al. 2008, Doerflinger et al. 2014).	Some strains are resistant to metronidazole and secnidazole (De Backer et al. 2010, Petrina et al. 2017).
*Mobiluncus mulieris*	Limited adherence to ME-180 cells and can be outcompeted by *L. crispatus* (Machado et al. 2013b). Does not form a biofilm (Patterson et al. 2010).	Is not cytotoxic (Patterson et al. 2010). Has proteolytic activity through the production of proline aminopeptidase (Schoonmaker et al. 1991).	Not described.	Increases inflammation through flagella-mediated TLR5 activation (McKenzie et al. 2021)	Some strains are resistant to metronidazole (Spiegel 1987).
*Sneathia amnii*	Can adhere to a ME-180 cervical cell line and encodes for a fibronectin-binding protein (Harwich et al. 2012).	Expresses the cytotoxin CptA (Gentile et al. 2020), and encodes for multiple putative invasins (Harwich et al. 2012). Produces polyamines (Łaniewski and Herbst-Kralovetz 2021).	Its genome encodes for a potential *O*-sialoglycoprotein endopeptidase. Can degrade glycogen (Harwich et al. 2012).	Induces a proinflammatory response in vaginal epithelial cells (Anahtar et al. 2015) and in a human 3D cervical cell model (Łaniewski and Herbst-Kralovetz 2021).	Sensitive to metronidazole (Harwich et al. 2012).

### *Hoylesella timonensis* (formerly *Prevotella timonensis*)

Different *Prevotella* spp. including *P. bivia* and *P. amnii* have been implicated in the pathogenesis of BV. Some of these bacteria are closely related with highly similar 16S rRNA genes, which has hampered classification in the past (Ilhan et al. 2020, Hitch et al. 2022). *Hoylesella timonensis*, a Gram-negative bacterium often present in women with BV (Srinivasan et al. 2012, Gao et al. 2022), was previously classified as *Prevotella timonensis*, but has recently been reassigned to the new genus *Hoylesella* based on phylogenetic analysis (Hitch et al. 2022). The pathogenic potential of *H. timonensis* has been relatively underexplored in comparison to *G. vaginalis*, but more publications are emerging that emphasize the unique contributions of *H. timonensis* to BV. Similar to *G. vaginalis, H. timonensis* can adhere to the vaginal epithelium (Segui-Perez et al. 2024). It can form a sparse biofilm and was shown to induce microvilli elongation in a 3D-endometrial cell model (Ilhan et al. 2020). It remains to be determined if *H. timonensis* can displace lactobacilli as has been demonstrated for *G. vaginalis*, but these traits suggest that *H. timonensis* could have the capacity to be an early colonizer in BV.

The *H. timonensis* genome does not encode any evident toxin genes. An important aspect of *H. timonensis* virulence potential seems to be its extensive repertoire of carbohydrate-active enzymes (CAZymes). *Hoylesella timonensis* encodes a broad array of mucin-degrading enzymes, including fucosidases and sialidases (Segui-Perez et al. 2024). The bacteria express two highly active GH33 sialidases, NanH1 and NanH2 and these *H. timonensis* enzymes are highly abundant in clinical BV samples as detected by metagenomic and metatranscriptomic analysis (Pelayo et al. 2024). The *H. timonensis* NanH1 and NanH2 enzymes effectively remove sialic acids from mucin substrates and the vaginal epithelial glycocalyx (Segui-Perez et al. 2024). Interestingly, similarly to *G. vaginalis* NanH3, the *H. timonensis* sialidases are also active at low pH, indicating that they potentially remain active in *Lactobacillus*-dominated communities (Pelayo et al. 2024). The high capacity of these bacteria to degrade mucus perhaps explains the association of *H. timonensis* with thinned vaginal discharge (Coleman and Gaydos 2018). In addition to the sialidases and fucosidases, *H. timonensis* encodes a broad spectrum of other CAZymes, including α-glucosidases that allow the bacteria to use glycogen as a sole carbon source (Jenkins et al. 2023, Segui-Perez et al. 2024).

*In vitro* studies indicate that *H. timonensis* does not induce production of proinflammatory cytokines by vaginal and endocervical cells (Ilhan et al. 2020, Segui-Perez et al. 2024). In contrast, other vaginal *Prevotella* spp., such as *P. amnii* and *P. bivia*, do induce secretion of IL-1α, IL-1β, and IL-8 from human vaginal epithelial cells (Anahtar et al. 2015, Gosmann et al. 2017). When in contact with dendritic cells, *H. timonensis* induces a strong proinflammatory response that is more significant than *G. vaginalis*. When T cells were exposed to *H. timonensis*, a T helper 1 skewing was observed that might be associated with enhanced susceptibility to STIs and preterm birth in the presence of *H. timonensis* (van Teijlingen et al. 2020). In conclusion, once *H. timonensis* establishes itself in an anaerobic vaginal microenvironment, it adheres efficiently to the epithelium, is well-adapted to utilizing glycogen as an energy source and can degrade the mucus layer, which may provide nutrients and adhesion sites for other bacteria. Its ability to control inflammatory responses by the epithelium and immune cells most likely contributes to long-term colonization of the vaginal environment.

### Prevotella bivia

*Prevotella bivia* is another member of the *Prevotella* clade and has been implicated in the pathogenesis of BV by multiple studies (Smayevsky et al. 2001, Muzny et al. 2018). Like *H. timonensis, P. bivia* is capable of forming a sparse biofilm in a 3D-endometrial cell model (Ilhan et al. 2020), but it does not strongly adhere to the cervicovaginal epithelium (Segui-Perez et al. 2024). Once *G. vaginalis* has established a biofilm, *P. bivia* can join in, suggesting that *P. bivia* may be a secondary colonizer (Machado et al. 2013, Castro et al. 2021). *Prevotella bivia* produces ammonia, which promotes the growth of *G. vaginalis* (Pybus and Onderdonk 1997), and the synergistic relationship between *P. bivia* and *G. vaginalis* likely contributes to the establishment of polymicrobial colonization that is characteristic of BV.

*Prevotella bivia* exhibits several enzymatic activities that may contribute to its pathogenic potential. It produces collagenase, which may promote the detachment of vaginal epithelial cells (Doust and Mobarez 2004, Łaniewski and Herbst-Kralovetz 2021). *Prevotella bivia* can grow on glycogen, but its mucin-degrading capacity is not as extensive as *H. timonensis* (Segui-Perez et al. 2024). However, *P. bivia* does express active fucosidases and sialidases *in vitro* and in mouse models (Gilbert et al. 2019, Segui-Perez et al. 2024). The *P. bivia* genome encodes two GH29 fucosidase genes and one GH33 sialidase gene (NanH). Unlike *H. timonensis*, the *P. bivia* enzymes are not active at the vaginal epithelial glycocalyx (Segui-Perez et al. 2024). These findings indicate that the differential levels of sialidase activity and substrate specificity among *Gardnerella, Hoylesella*, and *Prevotella* spp. are important characteristics that distinguish these bacterial species. Another property of *P. bivia* is the production of polyamines, such as putrescine, spermidine, and cadaverine, which are compounds that cause the malodor that can be a characteristic of BV and contribute to an increased vaginal pH (Nelson et al. 2015, Łaniewski and Herbst-Kralovetz 2021).

The inflammatory potential of *P. bivia* in the cervicovaginal environment remains unclear. *Prevotella bivia* did not induce a proinflammatory response or cytotoxicity in several studies (Doerflinger et al. 2014, Ilhan et al. 2020, Segui-Perez et al. 2024), but was reported to induce IL-6 and IL-8 expression in another study with cervical epithelial cells (Gosmann et al. 2017). It remains to be investigated if these differences are a result of strain variation between different studies. Overall, *P. bivia* does not appear to be an initial colonizer of the vaginal epithelium. However, once it integrates into the *G. vaginalis* biofilm, it supports the growth of *G. vaginalis* and expresses sialidases and collagenases, contributing to the epithelial damage that is characteristic of BV.

### *Fannyhessea vaginae* (formerly *Atopobium vaginae*)

*Fannyhessea vaginae*, previously known as *Atopobium vaginae*, is a facultative anaerobic Gram-positive commonly found in women with BV (Burton et al. 2004, Ferris et al. 2004, De Backer et al. 2007). *Fannyhessea vaginae* is often found alongside *G. vaginalis*, and the cooccurrence of these bacteria has been linked to adverse pregnancy outcomes, including miscarriage and preterm birth (Menard et al. 2010, Bretelle et al. 2015). In a competitive setting, *L. crispatus* can outcompete *F. vaginae* for attachment to vaginal epithelial cells (Machado et al. 2013) and a preexisting biofilm seems to be necessary for successful *F. vaginae* colonization (Hardy et al. 2016, Castro et al. 2019, 2020). *Fannyhessea vaginae* elicits a robust epithelial proinflammatory response in cervicovaginal epithelial cells, characterized by elevated levels of IL-6, IL-8, CCL-20, and TNFα (Trama et al. 2007, Libby et al. 2008, Doerflinger et al. 2014). In conjunction with other anaerobic bacteria, *F. vaginae* also increases paracellular permeability in ectocervical cells (Hinderfeld et al. 2019). Taken together, *F. vaginae* is an important secondary colonizer that can exacerbate BV by creating an inflammatory environment that contributes to epithelial disruption and recruitment of immune cells.

### *Mobiluncus* spp.

*Mobiluncus* bacteria are Gram-positive anaerobes of which several species have been associated with BV (Fredricks et al. 2005, Menard et al. 2008, Oakley et al. 2008). With respect to adherence and competition with healthy lactobacilli, it was shown that *M. mulieris* was able to displace *L. crispatus* adhered to glass slides (Machado et al. 2013) and the authors speculate that this *Mobiluncus* spp. may secrete soluble factors that displace lactobacilli. Contrary to *G. vaginalis, M. mulieris* did not form a biofilm on wells treated with polystyrene (Patterson et al. 2010), adherence to uterine cells was limited, and could be outcompeted by *L. crispatus* in one study (Machado et al. 2013)*. Mobiluncus mulieris* is not cytotoxic (Patterson et al. 2010), but it has been shown to have proteolytic activity through proline aminopeptidase (Schoonmaker et al. 1991). *Gardnerella vaginalis* grows faster in the presence of *M. mulieris*, which might be mediated by the effect of *Mobiluncus* peptides on *Gardnerella* (Machado et al. 2013). These studies suggest that *M. mulieris* contributes to BV progression by interacting with and promoting an environment that positively affects the growth of BVAB.

### *Sneathia* spp.

*Sneathia* spp., such as *S. amnii*, are often found in BV and have been associated with preeclampsia, spontaneous abortion, postpartum bacteremia, as well as with sexually transmitted diseases and cervical cancer (Shukla et al. 2002, Fredricks et al. 2007, Srinivasan and Fredricks 2008, Ling et al. 2010, Nawrot et al. 2010). *Sneathia* spp. have been isolated from amniotic fluid and its presence can lead to inflammation and cause pregnancy complications (DiGiulio et al. 2008, Han et al. 2009). In a 3D cervical cell model, infection with *S. amnii* was associated with the presence of clue cells (Łaniewski and Herbst-Kralovetz 2021). It was also reported that *S. amnii* can damage the fetal membranes, and is possibly related to the expression of the cytopathogenic toxin CptA (Gentile et al. 2020). Like *P. bivia, S. amnii* produces polyamines that are linked to altering the pH and malodor, which are associated with BV (Łaniewski and Herbst-Kralovetz 2021).

*Sneathia amnii* can adhere to ME-180 human cervical cancer cells and cause cell cytotoxicity, but does not form a biofilm (Harwich et al. 2012). Adhesion to and damage of the epithelium could be mediated by a putative adhesin, multiple invasins, a fibronectin-binding protein, and the hemolysin CptA that are encoded in the *S. amnii* genome (Harwich et al. 2012). *Sneathia amnii* infection leads to secretion of proinflammatory cytokines, increases oxidative stress, and alters cellular metabolism by cervicovaginal epithelial cell models (Anahtar et al. 2015, Łaniewski and Herbst-Kralovetz 2021). With respect to nutrient utilization, *S. amnii* can degrade glycogen and encodes a potential *O-*sialoglycoprotein endopeptidase that could be involved in the degradation of sialylated proteins (Harwich et al. 2012). This bacterial species also produces β-glucuronidases that degrade glycosaminoglycans found in the vaginal tissue (Collins et al. 2001), and high levels of these enzymes have been associated with aerobic vaginitis (Wang et al. 2016). Overall, *Sneathia* spp. harbor multiple virulence traits that allow these bacteria to spread within infected tissues in the female genital tract (FRT) and contribute to severe adverse effects of BV.

### *Megasphaera* spp.

*Megasphaera* spp., that can be subdivided in phylotype 1 (MP1) and phylotype 2 (MP2), are another type of bacteria associated with BV and pregnancy complications. *Megasphaera* spp. have been used in combination with other bacteria for molecular diagnosis of BV (Zozaya-Hinchliffe et al. 2008, Fethers et al. 2012, Datcu et al. 2014, Lennard et al. 2017). Both MP1 and MP2 were linked to elevated vaginal pH (Glascock et al. 2021). Women with high prevalence of MP1 and MP2 have an increased risk for HIV acquisition (McClelland et al. 2018, Sabo et al. 2020). MP2 was also associated with the sexually transmitted parasite trichomoniasis, more so than MP1 (Martin et al. 2013, Glascock et al. 2021). A study found MP1 in samples collected from the upper genital tract of women undergoing hysterectomy (Mitchell et al. 2015), suggesting that MP1 may be capable of ascending from the vagina. In contrast to the BV-associated *G. vaginalis*, both *M. elsdenii* and *H. timonensis* induce dendritic cell activation and proinflammatory cytokines (van Teijlingen et al. 2020), suggesting that they might be involved in inducing inflammation. The potential involvement of *Megasphaera* spp. in epithelial cytotoxicity and mucus degradation has not yet been explored.

### BVAB

BVAB 1, 2, and 3 are vaginal bacteria in the *Clostridiales* order that have recently been identified and linked to BV (Fredricks et al. 2005, Marrazzo et al. 2008, Mitchell et al. 2009). The presence of BVAB 2 was associated with elevated vaginal pH, a hallmark of BV. BVAB bacteria seem to be linked to increased HIV-1 shedding (Mitchell et al. 2009), HPV prevalence, and progression of cervical intraepithelial neoplasia (Mitchell et al. 2009, Naidoo et al. 2022). More studies are needed to investigate the virulence traits of BVAB and unravel how these bacteria contribute to BV.

### Effects of healthy status and BV on viral infections

**Figure 1.**
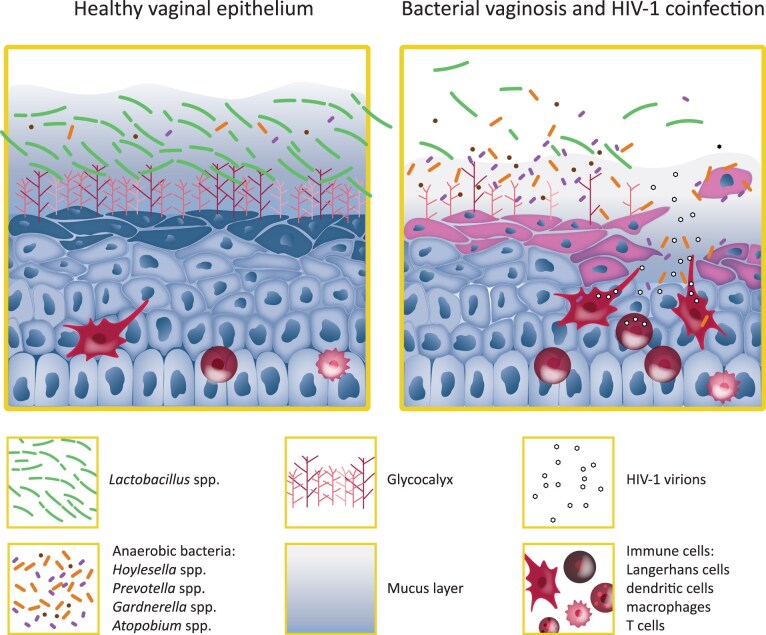
Bacterial interactions with the vaginal mucosal epithelium in health and disease. Schematic representation of the vaginal epithelium in a healthy state (left) and during BV with HIV-1 coinfection (right). The figure illustrates differences in microbiota composition, epithelial structure, and the presence of immune cells. Figure design by Soledad Cook-Ordonez.

The FRT is exposed to different (sexually transmitted) viral infections, including HIV-1, HPV, and HSV-2. BV patients are more susceptible to these infections. In this section, we describe how the composition of the vaginal microbiota and the BV-status affect the barrier functions of the CVM, the epithelial glycocalyx, epithelial cells and their connecting tight junctions (TJs) and interactions with immune cells that together determine the outcome of viral–host interactions (Fig. 1).

### Degradation of protective mucus affects virus infection

The vaginal mucosa is covered with a soluble mucus layer that mediates interactions with bacteria and viruses. A dense mucus “plug” is present in the cervix that limits access of microbes to the uterus. CVM mostly originates from the cervix and, together with antimicrobial compounds, protects the epithelium against chemical and biological agents (Lacroix et al. 2020). The CVM has been demonstrated to reduce the mobility of HIV-1 virions, particularly under acidic conditions (pH ∼4) (Nunn et al. 2015). This effect might be mediated by d-lactic acid produced by lactobacilli, which neutralizes the negative charge on the surface of HIV-1 virions, consequently increasing nonspecific interactions between the viral membrane and mucins (Lai et al. 2009, Nunn et al. 2015, Hoang et al. 2020). In BV, the mucosal defense system is compromised by the presence of elevated concentrations of mucin-degrading enzymes, such as sialidases, galactosidases, and sulfatases (Howe et al. 1999, Olmsted et al. 2003, Cauci et al. 2005, Moncla et al. 2015, 2016). CVM isolated from women with BV is thinner and has reduced viscosity, and the mobility of HIV-1 virions is increased in this diseased mucus (Hoang et al. 2020). The mucin-degrading ability of vaginal anaerobic bacteria is, therefore, an important virulence factor that reduces the ability of the CVM to trap viral particles.

### Compromising epithelial barrier function

Once the mucus layer is compromised, bacterial and viral particles come into closer contact with the vaginal epithelium. The vaginal glycocalyx is the carbohydrate-rich layer that coats the surfaces of vaginal epithelial cells and is formed by transmembrane mucins, such as MUC1 and MUC4 (Gipson et al. 1997, Hjelm et al. 2010). This glycocalyx interacts directly with vaginal bacteria and provides attachment sites for commensal lactobacilli and an additional protective layer for pathogens (Agarwal et al. 2022). Bacteria glycosylases secreted by BV-bacteria, such as sialidases and fucosidases, also degrade and modify the glycocalyx. Viral particles can traverse the epithelium via transcytosis, a process involving vesicular trafficking, the paracellular route that occurs through intercellular spaces, or direct infection of the epithelium and the release of novel virions. Studies have demonstrated that HIV-1 virions can undergo transcytosis through cervicovaginal and uterine epithelial cells (Asin et al. 2003, Gupta et al. 2013, Micsenyi et al. 2013), leading to the subsequent transmission of viral particles to CD4+ T cells (Asin et al. 2003, Stoddard et al. 2007, Micsenyi et al. 2013). BVAB such as *G. vaginalis* and *S. amnii* express the toxins vaginolysin and CptA, which induce cytotoxicity and release of cells from the epithelium. These so-called “clue cells” are coated with bacteria and used for BV diagnosis in the clinic. Cytotoxicity and clue cell formation contribute to tissue disruption (Cook et al. 1989), which could facilitate viral access deeper into the mucosa.

TJs, composed of transmembrane proteins, such as occludin, claudins, and zonula occludens, seal the intracellular space between neighboring epithelial cells. When intact, TJs form a selective barrier that restricts the passage of viral particles across the cervicovaginal and endocervical epithelial layers (Blaskewicz et al. 2011, Carias et al. 2013). However, under inflammatory conditions, such as bacterial and/or viral infections, TJ complexes become more open, allowing the migration of immune cells and resulting in increased permeability of the tissue (Tugizov 2021, Citi et al. 2024). Interestingly, immune cells such as dendritic cells and Langerhans cells, express TJ proteins that may allow them to open the paracellular space in a zipper-like manner and migrate through the epithelium (Rescigno et al. 2001, Ichiyasu et al. 2004, Zimmerli and Hauser 2007, Kubo et al. 2009). Anaerobic BVAB can modulate TJs and compromise the integrity of the vaginal epithelium through the production of cytotoxins (Patterson et al. 2010, Gilbert et al. 2019, Anton et al. 2022). They also induce the production of proinflammatory cytokines that alter TJ proteins, leading to epithelial cell damage and exfoliation (Hedges et al. 2006, Capaldo et al. 2014, Plesniarski et al. 2021). For intestinal pathogens such as *Clostridium perfringens* and *Campylobacter jejuni*, direct targeting of TJs with toxins and proteases has been described (Freedman et al. 2016) (Sharafutdinov et al. 2024). Whether BVAB also target TJs is currently not reported and remains to be investigated. Overall, a compromised epithelial barrier leads to enhanced contact of Langerhans cells and DCs with bacteria and/or viruses, thereby increasing the chance of successful viral infection and transmission.

### Roles of different immune cells in HIV-1 infection

The repertoire of immune cells present in the mucosa plays an essential role in the potential success of incoming viruses. Langerhans cells and CD4+ T cells are the initial immune cells that encounter HIV-1 in the lower genital tract (Hladik et al. 2007). Langerhans cells, predominantly present in the stratified squamous nonkeratinized epithelium, efficiently capture and internalize HIV-1 (Valladeau et al. 2000, Turville et al. 2002, Hladik et al. 2007). Once internalized, HIV-1 is targeted into specialized vesicles called Birbeck granules that are unique to Langerhans cells, where the virus is degraded via autophagy, preventing HIV-1 infection and transmission to susceptible CD4 + T cells (de Witte et al. 2007a, Ribeiro et al. 2016). Through this mechanism, Langerhans cells are protective against HIV-1 infection and prevent transmission to CD4 + T cells. Dendritic cells (DCs) are also present in the submucosal lamina propria layer and can detect and respond to HIV-1. DCs can capture HIV-1, but in contrast to Langerhans cells, do not protect against HIV-1 infection (Geijtenbeek et al. 2000a, b, Kwon et al. 2002, Shen et al. 2014). DCs that have captured and internalized HIV-1 can migrate to draining lymph nodes and transmit the virus to CD4 + T cells, thereby facilitating viral spread (Geijtenbeek et al. 2000a, Cameron et al. 1992, Weissman et al. 1995, Granelli-Piperno et al. 1999, Kwon et al. 2002, Shen et al. 2014). Macrophages that are present in submucosal vaginal tissue are also permissive for HIV-1 infection (Shen et al. 2009, 2011). Overall, recent studies suggest that during BV, the repertoire of immune cells present in the vaginal lamina propria is altered, which contributes to enhanced susceptibility to viral infections such as HIV-1.

### Proinflammatory immune activation and recruitment of immune cells

In addition to directly compromising epithelial barrier functions, the inflammation induced by BV-associated anaerobic bacteria can promote the production of proinflammatory cytokines and immune cell recruitment (Bleul et al. 1997, Hladik et al. 1999, McKinnon et al. 2011). Several BVAB, such as *G. vaginalis, F. vaginae, S. amnii*, and *M. mulieris* induce inflammation and lead to an increased proinflammatory response (Libby et al. 2008, Doerflinger et al. 2014, McKenzie et al. 2021, Łaniewski and Herbst-Kralovetz 2021, Anton et al. 2022). Proinflammatory responses are required to attenuate BV but also affect susceptibility to viral infections by attracting and activating target immune cells, as well as altering the function of immune cells. Under noninflammatory conditions, immature Langerhans cells inhibit HIV-1 infection and dissemination by capturing and degrading virions (de Witte et al. 2007a, Valladeau et al. 2000). Under inflammatory conditions, the antiviral capabilities of activated Langerhans cells are compromised, making them susceptible to HIV-1 infection and transmission to target cells (de Witte et al. 2007a, De Jong and Geijtenbeek 2009). BV is also linked to increased levels of proinflammatory cytokines that can lead to the recruitment and activation of immune cells including CD4 + T cells into vaginal tissue. The influx of T cells into the vaginal lamina propria results in enhanced susceptibility to HIV-1 due to proximity to the virus (Arnold et al. 2016). Furthermore, the number of activated target CD4 + T cells in genital mucosa is enhanced in germ-free mice following intravaginal colonization with BV-associated *P. bivia* (Gosmann et al. 2017). In addition, when DCs are exposed to BVAB and cocultured with T cells, *H. timonensis* skews CD4 + T cells toward a T helper 1 (Th1) response (van Teijlingen et al. 2020). A strong Th1 response can lead to uncontrolled inflammation and is typically associated with preterm birth (van Teijlingen et al. 2020). Overall, BVAB promote inflammation, compromise epithelial barrier functions, and alter immune responses, consequently increasing susceptibility to viral infections.

### Direct effects of BVAB on viral uptake by immune cells

Recently, we have discovered that BVAB have a direct impact on viral susceptibility by immune cells, specifically for HIV-1. Interestingly, *H. timonensis* enhances HIV-1 uptake by vaginal Langerhans cells, where the virions are protected from autophagy-mediated degradation and remain infectious for several days (Van Teijlingen et al. 2022). Furthermore, the enhanced viral uptake by Langerhans cells leads to enhanced transmission to susceptible HIV-1 target cells (Van Teijlingen et al. 2022). Moreover, exposure to *H. timonensis* also enhances HIV-1 uptake by DCs and CD4 + T cells (van Teijlingen et al. 2024, van Smoorenburg et al. 2025). In primary DCs, the increase in uptake results in enhanced transmission to target cells (van Smoorenburg et al. 2025). In vaginal CD4 + T cells, the *H. timonensis*-induced uptake results in productive infection of CD4 + T cells (van Teijlingen et al. 2024). This demonstrates that in addition to the indirect effects described above, a BV-associated bacterium can also directly affect the viral susceptibility of immune cells. The observation that multiple immune cells are affected by *H. timonensis* indicates that the underlying mechanism of enhanced uptake may not be restricted to a specific cellular receptor. Which specific virulence factor or property of *H. timonensis* is responsible for the enhanced viral uptake remains to be identified.

### BVAB affect HPV and HSV-2 susceptibility

Besides enhancing susceptibility to HIV-1, BVAB have also been linked to an increased risk of acquiring the STIs, HPV, and HSV-2 (Cherpes et al. 2005, Kaul et al. 2007, Nagot et al. 2007, Gillet et al. 2011, Moscicki et al. 2012, Łaniewski et al. 2018). An increase in vaginal anaerobic bacterial species is observed in HPV-infected women (Shannon et al. 2017). Furthermore, HPV infection has been significantly associated with the presence of *F. vaginae* (Di Paola et al. 2017) and BVAB bacteria (Naidoo et al. 2022). In addition, sialidases produced by *G. vaginalis* have been implicated in HPV persistence in cervical infections (Di Paola et al. 2017, Novak et al. 2023), although underlying molecular mechanisms remain to be investigated. Women who acquired an HPV infection show decreased numbers of Langerhans cells in the FRT (Jimenez-Flores et al. 2006), but women who in addition cleared the HPV infection, show increased numbers of endocervical LCs (Shannon et al. 2017). Both Langerhans cells and DCs interact with HPV and can facilitate viral spread (de Witte et al. 2007b, Yan et al. 2004, Bousarghin et al. 2005). In addition, CD4 + T cells can also interact with HPV (Williams et al. 2002), although no recruitment of CD4 + T cells is observed in HPV-infected women (Shannon et al. 2017).

In contrast to HPV, an increased number of activated mucosal CD4 + T cells as well as an enhanced number of cervical DCs are observed in the FRT during an HSV-2 infection (Rebbapragada et al. 2007, Shannon et al. 2014). Furthermore, an HSV-2 infection enhances the risk of acquiring an HIV-1 infection (Wald and Link 2002), which could in part be explained by HSV-2 affecting the antiviral properties of LCs as well as by the enhanced presence of HIV-1 target cells (Rebbapragada et al. 2007, de Jong et al. 2010). Altogether, different immune cells in the vaginal tissue play an essential role in establishing homeostasis and providing an additional line of defense against viral infections. Although more research is required, it is plausible that the same mechanisms by which BVAB enhance the risk of HIV-1 infection may apply to HPV or HSV-2 infections. These mechanisms could include decreased protective effects from *Lactobacillus*-dominated vaginal microbiota, changes in mucus levels, enhanced epithelial permeability, induction of a proinflammatory response with enhanced immune cell recruitment, and direct immune modulation by bacteria.

## Concluding remarks

BV is a polymicrobial infection that significantly impacts women’s quality of life and reproductive health. BV is associated with an increased risk of acquiring STIs, including HIV-1, HPV, and HSV-2. Traditionally, research has focused on *G. vaginalis*, due to its numerous virulence factors and high prevalence in women with BV. However, BV is a polymicrobial condition, and species within the genera *Hoylesella, Prevotella, Fannyhessea, Mobiluncus*, and *Sneathia* also exhibit important virulence traits. Bacterial glycosidases, adhesion, the proinflammatory impact on the epithelium, and recruitment of immune cells are all key factors that increase the risk of viral infection. More insight into the contributions of individual vaginal anaerobic bacteria and their synergistic effects that lead to BV and increased susceptibility to viral infections is necessary to develop new therapeutic interventions.
